# Physical Activity and Cardiovascular Disease in Japan: The Jichi Medical School Cohort Study

**DOI:** 10.2188/jea.JE20090051

**Published:** 2010-05-05

**Authors:** Yosuke Shibata, Shinya Hayasaka, Tomoyo Yamada, Yasuaki Goto, Toshiyuki Ojima, Shizukiyo Ishikawa, Kazunori Kayaba, Tadao Gotoh, Yosikazu Nakamura

**Affiliations:** 1 Department of Community Health and Preventive Medicine, Hamamatsu University School of Medicine, Hamamatsu, Shizuoka, Japan; 2 Department of Research, Japan Health Research Institute, Tokyo, Japan; 3 Division of Community and Family Medicine, Center for Community Medicine, Jichi Medical University, Shimotsuke, Tochigi, Japan; 4 School of Health and Social Services, Saitama Prefectural University, Koshigaya, Saitama, Japan; 5 Department of Public Health, Jichi Medical University, Shimotsuke, Tochigi, Japan

**Keywords:** motor activity, cardiovascular diseases, stroke, cohort studies, Japan

## Abstract

**Background:**

Many studies have reported an association between physical activity and cardiovascular disease (CVD); however, the effect of physical activity remains controversial. Few such studies have been conducted in Japan. Therefore, we examined the relationship between physical activity and death from CVD using prospective data from a Japanese population.

**Methods:**

From a prospective cohort study that comprised 12 490 participants, data from 9810 were analyzed. From April 1992 through July 1995, a baseline survey was conducted in 12 communities in Japan. The participants were followed up until December 2005. Physical activity was assessed using the physical activity index (PAI). PAI scores were grouped in quartiles: Q1 was the lowest PAI quartile and Q4 was the highest. Hazard ratios (HRs) for death from CVD, stroke, and myocardial infarction (MI) were calculated for all PAI quartiles.

**Results:**

The mean follow-up period was 11.9 years, during which time 194 participants died of CVD. With Q1 as the reference, the HRs for death from CVD in Q2, Q3, and Q4, were 0.62 (95% confidence interval, 0.40–0.98), 0.53 (0.31–0.88), and 0.40 (0.22–0.73), respectively, in men, and 0.71 (0.38–1.32), 0.52 (0.26–1.04), and 0.48 (0.22–1.05), respectively, in women. The HRs for death from CVD subtypes were similar but not statistically significant.

**Conclusions:**

Among a Japanese population, physical activity was associated with a decreased risk of death from CVD. However, more evidence is needed to elucidate the relationships between physical activity and CVD subtypes.

## INTRODUCTION

Cardiovascular disease (CVD) is the leading cause of death in Japan, and was responsible for more than 300 000 deaths in 2007.^1^ Most cases of CVD are patients with stroke or coronary heart disease (CHD). Although the incidence of stroke has been decreasing internationally, it remains a major cause of hospitalization and long-term disability in most developed countries.^2^ The 2 major causes of stroke are atherosclerosis of the intracranial or extracranial vessels and high blood pressure.^3^ Physical activity has been included in the search for preventive strategies because it can lower blood pressure, increase insulin sensitivity, elevate high-density lipoprotein cholesterol levels, and improve endothelial function.^4^^–^^6^ Several cohort studies have confirmed the overall benefit of physical activity in reducing the morbidity and mortality of stroke^7^^–^^9^; however, a dose-response relationship between physical activity and risk for stroke has not been clearly demonstrated. Some studies have shown a monotonic decrease in mortality with increasing physical activity,^10^ but others have noted a U-shaped association or no association at all.^11^ Many studies have found a relationship between physical activity and CHD.^12^^–^^14^ Yu et al reported that heavy physical activity decreased the risk of CHD.^13^ However, Lee et al reported that heavy physical activity was not associated with CHD risk.^14^ As is the case with stroke, a dose-response relationship between physical activity and the risk of CHD is yet to be confirmed.

Many population-based cohort studies of physical activity and CVD risk have been conducted in Western countries, but few have been undertaken in Japan.^15^^,^^16^ Noda et al reported that physical activity reduced the risk of CVD.^15^ However, Nakayama et al reported that physical activity did not significantly reduce the risk of CVD.^16^ Here, we examined the relationship between physical activity and death from CVD and its subtypes using data from a prospective population-based cohort study conducted in Japan.

## METHODS

### Study population

The Jichi Medical School (JMS) Cohort Study is a prospective population-based study in Japan. Its primary objective is to identify risk factors for atherosclerosis in participants from 12 rural communities. Mass screening examinations for cardiovascular disease have been conducted under the direction of the health and medical service law for the aged, and it was the system established by this law that was used for data collection. The baseline data were obtained between April 1992 and July 1995. A total of 12 490 individuals (4911 men and 7579 women) participated in the mass screening examination. In the present analysis, we analyzed physical activity index (PAI) data from 9810 individuals (3856 men and 5954 women) who had no history of stroke or myocardial infarction (MI) at baseline. Participants were followed up until the end of 2005. The details of the JMS cohort study have been reported elsewhere.^17^

### Measurements

Systolic blood pressure (SBP) and diastolic blood pressure (DBP) were measured with a fully automated sphygmomanometer, BP203RV-II (Nippon Colin, Komaki, Japan). Serum total cholesterol (TC) was measured by an enzymatic method. Body mass index (BMI) was calculated as weight in kilograms divided by the square of the height in meters. Information regarding medical history and lifestyle was obtained using a questionnaire to confirm whether or not the participant was taking medication and to determine the existence of previous or concurrent illnesses.

### Physical activity assessment

Physical activity was assessed at the baseline examination with the use of the Framingham Study Questionnaire,^18^ which was administered in an interview conducted by a trained reviewer.^19^ Information obtained included the average duration of sleeping, working, and leisure activities during a normal weekday. The PAI was estimated by calculating the weighted sum of hours spent at 5 levels of activity using the following scores: 1.0 for a basal level of activity such as sleeping and resting, 1.1 for a sedentary level of activity such as working in a sitting position, 1.5 for slight activity such as working in a standing position, 2.5 for a moderate level of activity such as gardening, and 5.0 for a heavy level of activity such as shoveling. For example, “a subject who sleeps continually would receive a score of 24. An office worker with no outside exercise would have a score of 27 (8 hours at a basal level, 12 hours at a sedentary level, and 4 hours at a slight level of activity). A laborer who engaged in heavy activity could have a score of 42 (8 hours at a basal level, 8 hours at a sedentary level, 2 hours at a slight level, 3 hours at a moderate level, and 3 hours at a heavy level of activity)” according to the study criteria.^18^

### Diagnostic criteria

Information on deaths was obtained from death certificates, which were collected at the respective local public health centers with permission from the Japanese Ministry of General Affairs and the Ministry of Health, Labour and Welfare. We used the International Classification of Diseases, Tenth Revision (ICD-10) for diagnostic criteria: CVD comprised stroke (I60–I69), myocardial infarction (MI) (I01–I09, I21–I22, I27, I30–I49, I50, I51–I52), and others (others within I00–I99). Cases of sudden death due to an unidentified cause were not included in the category of CVD. Participants who moved out of their communities during the observation period were followed up until the date of their departure. Data on emigration of the study participants were obtained every year from their municipal governments.

### Statistical analysis

Subjects were divided into quartiles by PAI: Q1 was the lowest PAI quartile, and Q4 was the highest. The ranges of PAI scores were 24.0 to 28.8, 28.9 to 34.1, 34.2 to 38.3, and ≥38.4 for quartiles Q1 through Q4, respectively, for men, and 24.0 to 28.0, 28.1 to 30.2, 30.3 to 33.8, and ≥33.9, respectively, for women.

For the comparison of baseline characteristics, we used 1-way analysis of variance for continuous variables and the chi-square test for categorical variables. Cox’s proportional hazards model was used to calculate the hazards ratios (HRs) with 95% confidence intervals (CIs) for death from CVD according to PAI, after adjusting for potential confounding factors such as geographic area and age (model 1) and geographic area, age, SBP, BMI, total cholesterol (TC), current smoking status, current drinking status, education, job, and history of diabetes mellitus (model 2). Statistical analysis was performed using SPSS software version 15.0 (SPSS Japan Inc.).

### Ethics

This study was approved by the Institutional Review Board of Jichi Medical School. Written informed consent was obtained from each participant.

## RESULTS

The total number of observed person-years was 116 844, and the mean follow-up period ± standard deviation (SD) was 11.9 ± 2.4 years. There were 194 deaths from CVD (105 men and 89 women) during the study period.

The baseline characteristics of the study participants, stratified by PAI, are shown in Tables 1 and 2. Descriptive parameters are shown as mean, standard deviation, or proportion. Among quartiles, there were significant differences in age, BMI, TC, current smoking status, current drinking status, education, job, and history of diabetes mellitus.

**Table 1.** Characteristics of male participants at baseline, according to physical activity index

	Quartile 1 (≤28.8)	Quartile 2 (28.9–34.1)	Quartile 3 (34.2–38.3)	Quartile 4 (≥38.4)	*P*
Number of cases	979	975	989	974	
Age (y)	54.9 (13.1)	57.0 (12.1)	54.9 (11.2)	53.2 (11.4)	<0.01
Systolic blood pressure (mm Hg)	131.3 (20.1)	131.5 (20.3)	131.5 (19.9)	129.4 (20.4)	0.06
Body mass index (kg/m^2^)	23.1 (3.0)	23.0 (2.9)	22.8 (2.8)	22.7 (2.7)	<0.01
Serum total cholesterol (mg/dL)	188.3 (35.8)	185.7 (34.6)	183.6 (33.2)	182.9 (33.6)	<0.01
Current smoker (%)	52.8%	47.1%	52.7%	51.0%	0.04
Current drinker (%)	72.9%	74.8%	77.0%	78.1%	0.03
Education ≥15 years (%)	64.1%	56.8%	56.0%	49.2%	<0.01
Job in farming/forestry/fishery (%)	14.1%	31.7%	45.4%	57.1%	<0.01
History of diabetes mellitus (%)	6.8%	5.6%	3.8%	4.6%	0.02
Number of cardiovascular disease deaths	39	28	24	14	
Deaths from stroke	19	11	13	8	
Deaths from myocardial infarction	13	14	10	6	
Deaths from other cardiovascular disease	7	6	1	0	

Q1 is the lowest quartile of physical activity; Q4 is the highest.

Data are expressed as a mean (standard deviation) for variables or as a percentage of the population.

One-way analysis of variance was used for continuous variables and the chi-square test for categorical variables.

**Table 2.** Characteristics of female participants at baseline, according to physical activity index

	Quartile 1 (≤28.0)	Quartile 2 (28.1–30.2)	Quartile 3 (30.3–33.8)	Quartile 4 (≥33.9)	*P*
Number of cases	1534	1514	1517	1531	
Age (y)	54.1 (12.8)	54.9 (11.5)	55.8 (10.6)	56.0 (9.7)	<0.01
Systolic blood pressure (mm Hg)	126.9 (21.3)	127.3 (21.8)	128.0 (20.3)	128.2 (20.2)	0.29
Body mass index (kg/m^2^)	23.0 (3.2)	23.1 (3.2)	23.1 (3.2)	23.0 (3.0)	0.82
Serum total cholesterol (mg/dL)	197.0 (35.5)	197.3 (35.2)	198.8 (34.3)	193.6 (33.8)	<0.01
Current smoker (%)	7.4%	6.4%	5.1%	4.1%	<0.01
Current drinker (%)	28.0%	26.8%	23.5%	23.0%	<0.01
Education ≥15 years (%)	52.6%	51.6%	48.3%	47.2%	<0.01
Job in farming/forestry/fishery (%)	5.3%	10.9%	14.6%	57.6%	<0.01
History of diabetes mellitus (%)	4.2%	3.0%	3.0%	2.5%	0.04
Number of cardiovascular disease deaths	40	21	16	12	
Deaths from stroke	22	12	6	6	
Deaths from myocardial infarction	16	8	10	5	
Deaths from other cardiovascular disease	2	1	0	1	

Q1 is the lowest quartile of physical activity; Q4 is the highest.

Data are expressed as a mean (standard deviation) for variables or as a percentage of the population.

One-way analysis of variance was used for continuous variables and the chi-square test for categorical variables.

The HRs for death from CVD are shown in Table 3. Using Q1 as reference, the HRs were adjusted for geographic area and age (model 1) and for multiple variables (model 2). Using model 1, the HRs for Q1 through Q4 were 1.00 (reference), 0.62 (95% CI, 0.40–0.98), 0.53 (0.31–0.88), and 0.40 (0.22–0.73), respectively, in men, and 1.00, 0.71 (0.38–1.32), 0.52 (0.26–1.04), and 0.48 (0.22–1.05), respectively, in women. A higher quartile was associated with a decreased HR for death from CVD (men, *P* < 0.01 for trend; women, *P* = 0.03 for trend), and the HRs for Q4 were lowest in both men and women. Even after multivariate adjustment (model 2), the inverse association remained.

**Table 3.** Hazard ratios (HRs) plus 95% confidence intervals (CIs) for death from cardiovascular disease, according to physical activity

	Model 1		Model 2	
		
	HR	95% CI	HR	95% CI
Men				
Q1 (≤28.8)	1.00		1.00	
Q2 (28.9–34.1)	0.62	(0.40–0.98)	0.66	(0.41–1.06)
Q3 (34.2–38.3)	0.53	(0.31–0.88)	0.65	(0.37–1.14)
Q4 (≥38.4)	0.40	(0.22–0.73)	0.48	(0.24–0.93)
*P* for trend	*P* < 0.01		*P* = 0.03	

Women				
Q1 (≤28.0)	1.00		1.00	
Q2 (28.1–30.2)	0.71	(0.38–1.32)	0.70	(0.36–1.35)
Q3 (30.3–33.8)	0.52	(0.26–1.04)	0.50	(0.24–1.06)
Q4 (≥33.9)	0.48	(0.22–1.05)	0.49	(0.21–1.15)
*P* for trend	*P* = 0.03		*P* = 0.05	

Q1 is the lowest quartile of physical activity; Q4 is the highest.

Model 1 was adjusted for geographic area and age.

Model 2 was adjusted for geographic area, age, systolic blood pressure, body mass index, serum total cholesterol, smoking, drinking, education, job, and history of diabetes mellitus.

*P* for trend was calculated by treating category scales as continuous variables.

We proceeded to examine deaths from stroke and MI separately in a secondary analysis, as shown in Table 4. Using model 1, the HRs for death from stroke for Q1 to Q4 were 1.00 (reference), 0.64 (95% CI: 0.33–1.25), 0.67 (0.33–1.36), and 0.59 (0.27–1.33), respectively, in men, and 1.00 (reference), 0.88 (0.39–2.01), 0.26 (0.07–0.94), and 0.54 (0.18–1.62), respectively, in women. The HRs for death from MI were 1.00 (reference), 0.63 (0.32–1.26), 0.52 (0.23–1.16), and 0.37 (0.14–0.97), respectively, in men, and 1.00 (reference), 0.59 (0.21–1.65), 0.98 (0.39–2.50), and 0.49 (0.14–1.70), respectively, in women. Again, even after multivariate adjustment (model 2), these associations remained. We also calculated HRs for death from CVD after excluding individuals who died within 2 years from baseline, and the results were similar to those of the primary analysis (data not shown).

**Table 4.** Hazard ratios (HRs) plus 95% confidence intervals (CIs) for death from stroke and myocardial infarction, according to physical activity

		Model 1		Model 2	
			
		HR	95% CI	HR	95% CI
Men					
Stroke	Q1 (≤28.8)	1.00		1.00	
	Q2 (28.9–34.1)	0.64	(0.33–1.25)	0.60	(0.29–1.25)
	Q3 (34.2–38.3)	0.67	(0.33–1.36)	0.75	(0.34–1.67)
	Q4 (≥38.4)	0.59	(0.27–1.33)	0.63	(0.24–1.60)
	*P* for trend	*P* = 0.21		*P* = 0.36	

Myocardial infarction	Q1 (≤28.8)	1.00		1.00	
	Q2 (28.9–34.1)	0.63	(0.32–1.26)	0.77	(0.37–1.61)
	Q3 (34.2–38.3)	0.52	(0.23–1.16)	0.75	(0.32–1.79)
	Q4 (≥38.4)	0.37	(0.14–0.97)	0.57	(0.20–1.61)
	*P* for trend	*P* = 0.03		*P* = 0.30	

Women					
Stroke	Q1 (≤28.0)	1.00		1.00	
	Q2 (28.1–30.2)	0.88	(0.39–2.01)	0.89	(0.38–2.12)
	Q3 (30.3–33.8)	0.26	(0.07–0.94)	0.20	(0.04–0.91)
	Q4 (≥33.9)	0.54	(0.18–1.62)	0.54	(0.17–1.74)
	*P* for trend	*P* = 0.07		*P* = 0.08	

Myocardial infarction	Q1 (≤28.0)	1.00		1.00	
	Q2 (28.1–30.2)	0.59	(0.21–1.65)	0.50	(0.16–1.56)
	Q3 (30.3–33.8)	0.98	(0.39–2.50)	0.95	(0.35–2.56)
	Q4 (≥33.9)	0.49	(0.14–1.70)	0.53	(0.14–2.03)
	*P* for trend	*P* = 0.47		*P* = 0.59	

Q1 is the lowest quartile of physical activity; Q4 is the highest.

Model 1 was adjusted for geographic area and age.

Model 2 was adjusted for geographic area, age, systolic blood pressure, body mass index, serum total cholesterol, smoking, drinking, education, job, and history of diabetes mellitus.

*P* for trend was calculated by treating category scales as continuous variables.

## DISCUSSION

We investigated the association between physical activity and death from CVD using data from the JMS cohort study, a prospective population-based study. Physical activity reduced the risk for death from CVD in both male and female participants. A PAI score in a higher quartile was associated with a decreased HR for death from CVD (men, *P* = 0.03 for trend; women, *P* = 0.05 for trend), and the beneficial effects persisted even after adjustment for potential confounding factors. In addition, we analyzed stroke and MI separately. Physical activity moderately reduced the risk of stroke in women, but did not significantly reduce this risk in men. Physical activity appeared to reduce the risk of MI in both sexes, but the effect was not significant.

Regarding the presence of a dose-response relationship, studies have reported an inverse association between physical activity and CVD risk in men^20^ and women.^21^ However, some studies have found no inverse association.^22^^,^^23^ The College Alumni Health study, which enrolled only women, found no such association.^22^ The Adventist Mortality study, which did not adjust for biomarkers such as blood pressure and cholesterol, found a U-shaped relationship.^23^ Potential confounding factors may partially account for these discrepancies. The present results are consistent with those of many previous studies, which show that physical activity significantly reduces the risk of CVD and that a higher level of physical activity is associated with a decrease in HRs.^24^^,^^25^

In secondary analyses, we examined the association between physical activity and death from stroke and MI. Our results showed that physical activity reduced stroke risk among women, but that this effect was not significant among men. Previous studies have shown that physical activity reduced the risk of stroke among both men and women.^10^^,^^11^ The most likely reason why the present study failed to find such an association is that the sample size was insufficient for analysis by detailed cause of death. Some studies reported that habitual exercise and infrequent physical activity, eg, once a week, decreased stroke risk.^15^^,^^26^ If we had collected such information, habitual exercise might indeed have been associated with stroke risk. Although ischemic, hemorrhagic, and subarachnoid strokes have different causes,^27^ previous studies have shown that physical activity reduces the risk for all 3 types of stroke.^9^^,^^10^^,^^25^ Additional studies of physical activity and stroke subtypes are needed in Asian countries.

Our results showed that physical activity did not significantly reduce the risk of MI in either sex. However, the number of deaths due to MI was lower than that due to stroke, because in Japan, unlike in most Western countries, the incidence of MI is lower than that of stroke.^28^ We believe that the low number of MI events observed in the present study may have affected the results. Therefore, in this study we cannot draw any conclusions as to the relationship between physical activity and CVD subtypes.

The observed reduction in the risk of death from CVD may be due to the fact that physical activity decreases platelet aggregation, increases insulin sensitivity, reduces weight, increases HDL cholesterol levels, and/or reduces blood pressure.^29^^,^^30^ Risk factors for stroke include hypertension, diabetes mellitus, body weight, and complex atherosclerotic processes. A multitude of physiological and psychosocial variables may influence the effects of physical activity on the cerebrovascular system.

Our study has several strengths. It is a large-scale population-based study that controlled for various potentially confounding factors. It was conducted in a Japanese population, for which few data have been reported, and reports from Asian countries are needed in order to add to the evidence already accumulated in Western countries. However, it does have several limitations. Physical activity was self-reported, and some misclassification of the level of activity is inevitable. Most physical activity questionnaires have been developed for and validated in men, and activities traditionally performed by women, such as housework, are recorded in less detail. Although Garcia-Palmieri et al. confirmed the reliability and validity of this questionnaire,^31^ its reliability and validity have been less well evaluated in Japan.^32^^,^^33^ However, to ensure that accurate information was obtained from all individuals, the questionnaire was administered in an interview conducted by a trained reviewer.^19^ In the present study, we were not able to adjust for some confounding factors, including diet. In addition, although we examined only CVD deaths, it may be that physical activity has different effects on CVD incidence and death. However, many studies have reported that physical activity reduced the risk of both incidence and death from CVD.^12^^,^^24^ Some participants might change their patterns of physical activity during follow-up. However, this would likely result in an underestimation of the association between physical activity and CVD risks. Our sample size was probably too small to reveal associations with regard to detailed cause of death. Although lifestyle differences among countries might affect assessment of PAI, our study subjects had a PAI almost identical to that of the subjects in the Framingham study, for which the PAI was developed. Most physical activity questionnaires have been developed for use in Europe and the United States. We need more assessments of physical activity from Asian countries. The final limitation of the present study was that some diseases under treatment at baseline might have affected the level of physical activity. However, in a separate analysis of HRs that excluded individuals who died within 2 years from baseline, the results were similar to those of the main analysis.

In conclusion, we observed an inverse association between physical activity and the risk of death from CVD among both men and women in Japan. However, more reports are needed from Asian countries to determine the relationship between physical activity and CVD subtypes.
